# Measuring the impact of a training intervention for early childhood centre staff on child development outcomes: Findings from a cluster randomized control field trial in rural Malawi

**DOI:** 10.1111/cch.12981

**Published:** 2022-02-09

**Authors:** Emma Jolley, Stevens Bechange, Mika Mankhwazi, Jenipher Mbukwa Ngwira, Rachel Murphy, Elena Schmidt, Paul Lynch

**Affiliations:** ^1^ Health and Disability Research Sightsavers Haywards Heath UK; ^2^ Health and Disability Research Sightsavers Uganda Kampala Uganda; ^3^ Sandi Thandiza Malawi Lilongwe Malawi; ^4^ Catholic University of Malawi Limbe Malawi; ^5^ Evidence, Research and Innovations Sightsavers Haywards Heath UK; ^6^ Inclusive Education, School of Education University of Glasgow Glasgow UK

**Keywords:** child development, child disability, inclusion, measurement, randomized trials

## Abstract

**Background:**

Evidence from low‐income settings around early education interventions that can improve young children's development is sparse, particularly with regard to the most marginalized children. This study used a two‐arm parallel cluster randomized control design to evaluate the impact of an adapted staff training programme on the developmental outcomes of children attending community‐based early learning centres in Thyolo district, rural Malawi.

**Methods:**

At baseline we randomly selected 48 centres, from each of which 20 children were randomly selected, although data from one centre was incomplete resulting in 932 children from 47 centres. Centres were randomly allocated to either the intervention or control arm. Twelve months later, follow‐up data were collected from 44 centres. At baseline and endline, community‐based childcare centre (CBCC) managers provided information about the centre, and parents/guardians provided information on the children, including the primary outcomes of age‐standardized development scores in the language and social domains, measured using the Malawi Developmental Assessment Tool. Children in the bottom 2.5 percentile of either domain were considered to have a delay; a third outcome variable, Any Delay, was developed to indicate children with a delay in either or both domains. Centre‐level mean scores were calculated, and linear regression models were constructed to assess differences between baseline and endline and between allocation groups.

**Results:**

Analysis of the difference between baseline and endline measures in the allocation groups shows a non‐significant reduction in delay associated with the study intervention across all domains. Adjustment for baseline characteristics within the CBCCs showed little impact on the magnitude of the observed effect, and the difference remained non‐significant.

**Conclusions:**

Despite no observed differences between allocation groups, the data did indicate a positive change in the intervention groups in both domains, particularly language. Community‐based early learning in Malawi holds tremendous potential for promoting inclusive development and learning.

**Key Messages**

- The study is one of few to have used a rigorous experimental design to assess the effectiveness of an early education intervention on the developmental outcomes of young children in a low‐income setting.
- Despite not observing statistically significant results, the findings point towards the intervention having a positive effect on children development, particularly in the language domain.
- The study findings are consistent with the only other study using a similar approach and suggest that community‐based early learning centres have huge potential for improving child development outcomes, particularly when coupled with interventions targeting parenting.

## 1 INTRODUCTION

Evidence from low‐ and middle‐income countries (LMICs) suggests that as many as 250 million children under the age of 5 are at risk of not reaching their developmental potential (Black et al., 2017). An estimated 53 million children globally have a developmental disorder with most of these children living in LMICs (Olusanya et al., 2018). Children at highest risk of developmental delay are girls, those living in extreme poverty, those with disabilities, refugees and children with multiple characteristics of disadvantage (Black et al., 2017). The implications of unmet developmental potential extend beyond the individual child, to their families, communities and broader societies (Richter et al., 2017). Early detection of these disabilities is vital with early interventions reducing social, behavioural and educational problems (Milner et al., 2019). This, in turn, can reduce future costs for society and enable children to flourish, learn and participate better (Lancaster et al., 2018). Although the impact of the Covid‐19 pandemic is not yet fully understood, it is likely that the developmental challenges faced by children in poorer settings will be aggravated, exacerbating the inequity observed in child development globally (Yoshikawa et al., 2020). Interventions to improve child development outcomes that are not only culturally appropriate and acceptable, but also effective for the most marginalized children, are vital to improve this situation (Pence & Marfo, 2008).

Early childhood development (ECD) encompasses the inputs necessary to help shape the lives of children aged 0 to 8 years to survive, thrive and secure solid foundation (Richter et al., 2019). The five key components of ECD are good health, adequate nutrition, responsive caregiving, security and safety and opportunities for early learning (World Health Organization, 2018). While the evidence based on health and nutrition has substantially increased in recent years, that around the consequences and impact of early learning, particularly in informal settings, continues to be scarce (Berlinski et al., 2009; World Health Organization, 2012).

Malawi is a low‐income country in southern Africa, with a well‐developed policy base for ECD, overseen by the Ministry of Gender, Community Development and Social Welfare (MoGCDSW), and delivered mainly in informal community‐based settings (Neuman et al., 2014; World Bank, 2015). Despite strong policy buy‐in, attendance is relatively low, particularly in rural locations where 46% of households fall into two lowest economic quintiles and where stunting in children is particularly severe (Mussa, 2014; National Statistical Office [Malawi] and ICF, 2017). ECD interventions are delivered in community‐based childcare centres (CBCCs) by volunteer caregivers who are usually paid in kind. Significant challenges with many CBCCs include high caregiver–children ratios, untrained staff and high staff attrition (Munthali et al., 2014). Staff training is provided through a small number of government‐recognized organizations, for example, Association of Early Childhood Development Malawi (AECDM); however, they are dependent on funding from international donors that can be sporadic and insufficient for the needs. This was confirmed in previously published data from an initial component of this study, where CBCC caregivers emphasized the importance of receiving additional competency training and skill development, particularly in teaching children with disabilities (Greenwood et al., 2020; Soni et al., 2020).

The objective of this study was to assess the impact of an adapted training programme, which integrated a disability inclusive resource pack into the early years curriculum, on children's developmental outcomes (Soni et al., 2020). Results of the baseline published earlier showed high prevalence of both developmental delays and disability, with a strong relationship between the two (Murphy et al., 2020).

## 2 METHODS

### 2.1 Design and setting

We undertook a two‐arm parallel cluster randomized control trial (CRCT) in Thyolo district of Southern Malawi. The study was implemented in accordance with the Consolidated Standards of Reporting Trials (CONSORT) checklist (Campbell et al., 2004).

### 2.2 Ethics

CBCC representatives provided consent to observe CBCC activities and individual caregivers provided written consent to be interviewed. Parents/guardians provided written consent on behalf of their children. The consent was witnessed in the case of illiterate individuals or those aged under 18 years.

The trial was approved by the National Committee on Research in the Social Sciences and Humanities, National Commission for Science and Technology, Malawi (P.02/16/83) and the Ethics Committee of the University of Birmingham, UK (ERN_15‐0048).

### 2.3 Participants

The District Social Welfare Office maintains a list of registered CBCCs, which served as our sampling frame. At baseline, 48 CBCCs were randomly selected from a pool of CBCCs meeting minimum criteria: located in Thyolo district, feeding programme in place, more than 20 children registered and regularly attending, minimum of two caregivers, minimum infrastructure (e.g., permanent location, water supply) and had not participated in an earlier CRCT funded by the World Bank (Ozler et al., 2016).

Within each CBCC, 20 children were randomly selected at both baseline and endline. Selection for participation at both time points was independent, and the children's data are not linked from baseline to endline. Children were eligible for selection if they were at least 2 years old, had been registered for at least 6 months and attended the centre regularly (at least four times a month).

### 2.4 Sample size

The sample size was calculated to detect a 10% change in the proportion of children whose developmental age is equal to their biological age (expected increase from 70% to 80%). Based on the 95% confidence interval (CI), 80% power, 10% non‐response and 50% variation between the clusters (7), we aimed to recruit 960 children (480 per arm) or 48 CBCCs with 20 children per CBCC.

### 2.5 Randomization

The 48 study CBCCs were randomized to two arms. Randomization was carried out by one of the co‐authors (S.B.) before the trial started, using Excel. The children were randomly selected on the day of data collection using the CBCC records. If there were 20 or fewer children available on the day, then all were enrolled. If there were more than 20 children, then a random selection approach was applied by writing all names on pieces of paper, placing in a bowl and drawing at random.

Given the nature of the intervention, it was not possible to blind participants or data collectors.

### 2.6 Interventions

The adapted 14‐day training programme was provided to caregivers from 24 intervention CBCCs. The training was implemented over 4 weeks in July 2017, with caregivers divided in two groups, to allow CBCCs to continue functioning during the training period. Training was facilitated by national trainers from AECDM, Magomero Community Development College and the MoGCDSW. An ‘Inclusion Resource Pack’ was developed by the University of Birmingham in collaboration with Chancellor College of the University of Malawi, international non‐governmental organization, Sightsavers and AECDM and focused on promoting the inclusion of children with disabilities in the daily activities of the CBCCs (Soni et al., 2020). Training covered the following topics: understanding of disability, inclusive games, early literacy and storytelling, well‐being and involvement, safety and risk management, early maths, inclusive environment, inclusion of CBCCs, identification of common types of disability and working with parents of children with disabilities. The team enhanced the basic 2‐week training for volunteer caregivers building on the local practices, knowledge and strengths that exist in ECD and collaborate with local training providers, community and ECD services (Soni et al., 2020).

In addition to the training, all intervention CBCCs received a basket of simple locally sourced materials (laminated alphabet and number cards, string, pegs, a locally made parachute, etc.) to facilitate learning and inclusion of children with disabilities.

Control centres received the training in November 2018, after the collection of the endline data.

### 2.7 Outcomes

The primary outcome was the proportion of children with age‐appropriate scores in (1) language and (2) social–emotional development. We used two of the four domains of the Malawi Developmental Assessment Tool (MDAT) to ascertain the scores; the tool was developed based on the age‐standardized development norms appropriate for children living in rural Malawi (Gladstone et al., 2010). Children carried out up to 68 tasks that are incrementally more difficult. The assessment was conducted in the presence of the parent/guardian who could provide some of the answers to routine‐based questions. Numerical developmental scores in both domains were transformed to binary indicators, identifying those scores that fell in the bottom 2.5th percentile of the age‐referenced data as representing an observed delay in that domain. A third binary outcome variable, any delay, measured the proportion of children who had delay in one or both domains.

### 2.8 Explanatory variables

In addition to the developmental scores, parents/guardians of the selected children answered questions about their child's sex, age and disability status. The latter was assessed using the UNICEF/Washington Group Child Functioning Module (CFM) that assesses functional difficulties in different domains including hearing, vision, communication/comprehension, learning, mobility and emotions (Loeb et al., 2017). There are two versions of the tool: one for children aged 2–4 years and one for children aged 5–17 years; and we used both according to the age of our participants.

Data on the CBCCs themselves were collected at baseline through an interview with the head/chairman of the CBCC or a senior caregiver. These included some basic information about the CBCC, its record keeping, curriculum and resources available. For the purpose of the analysis, four CBCC characteristics were considered: number of caregivers per CBCC, number of children per CBCC, availability of a regularly kept attendance register and availability of learning and play materials.

### 2.9 Data collection

Six data collectors with experience of working with young children in rural Malawi were provided a 5‐day training on survey approaches, ethics and tools prior to both baseline and endline data collection. The training included practical examples of collecting data from the parents, including children's abilities based on the MDAT tool. A Malawian master MDAT trainer participated to ensure data collectors were conducting the assessments in a standardized manner.

Data collectors worked in two teams of three to visit the CBCCs. Survey tools were programmed into a mobile phone app using Kobo Toolbox in English and Chichewa languages (Harvard Humanitarian Initiative, 2017). The data were synced with the cloud at the end of each day, and the data were regularly downloaded and checked. Data collectors were contacted immediately if any unusual patterns or anomalies were observed to check and verify inputs.

### 2.10 Statistical methods

Basic descriptive statistics were calculated to understand the range and distribution of responses. We examined baseline characteristics by allocation group to understand whether values were similar. We did not perform formal statistical tests to assess homogeneity as we believe the randomization process was robust.

Raw summary scores of the percentage of children with a delay for baseline and endline were calculated according to allocation group. CBCC‐specific differences from baseline to endline were calculated for each of the three outcomes, which resulted in a mean percentage difference. We used linear regression to assess the difference between the mean percentage difference in the allocation groups and the associated 95% CI.

Additional analyses were conducted to account for baseline values of the CBCCs that were considered a priori, as having potential influence on the outcomes. We did this through adjusting the linear regression models by including the appropriate baseline values and reviewing the new difference in mean percentage difference and associated 95% CIs.

## 3 RESULTS

### 3.1 Participant flow and recruitment

Baseline data were collected between December 2016 and May 2017 from 48 CBCCs selected for the study. Data for one CBCC were incomplete and excluded from the analysis resulting in 23 intervention and 24 control CBCCs at baseline.

**FIGURE 1:**
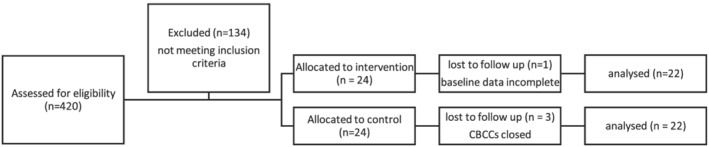
Flow chart of CBCC flow

Endline data were collected 1 year later, between May and July 2018. Three CBCCs, one in the intervention group and two in the control group, had closed since the baseline resulting in 44 CBCCs (22 per arm) with complete data (Figure 1). Analysis of our data to explore whether the CBCCs that closed differed from those that remained open indicated no differences between centre of child‐level characteristics (results not shown).

### 3.2 Baseline data

Summary statistics for 47 CBCCs at baseline are shown in Table 1 indicating similar mean number of children and caregivers per CBCC in the allocation groups. The proportion of CBCCs with attendance registers was slightly higher in the intervention group, while the proportion of CBCCs with learning and play materials was slightly higher in the control group.

**TABLE 1:** CBCC characteristics at baseline, by allocation group

	Control	Intervention	Total
CBCCs	24	23	47
Total number of caregivers employed	64 (2.7 per CBCC)	67 (2.9 per CBCC)	131
Total number of children registered	1,624 (67.7 per CBCC)	1,453 (63.2 per CBCC)	3,077 (65.5 per CBCC)
CBCC maintains child attendance register	16 (66.7%)	20 (87.0%)	36 (76.6%)
CBCC has learning/play materials (high score)	7 (29.2%)	4 (17.4%)	11 (23.4%)

Child‐level summary statistics are shown in Table 2. Child‐level data were available for 932 children at baseline and 881 at endline. At baseline, the mean age was 51.4 months, and 55.4% were girls; the age and sex profiles were similar in the allocation groups. At endline, the intervention CBCCs had more girls (63.4% vs. 54.2%), and the children were slightly older than in the control CBCCs (54.8 vs. 51.5 months). Over 10% of children at baseline were regarded as having a disability using the CFM tool with a slightly higher proportion in the intervention group. At endline, 6% of children had a disability with no difference between the groups.

**TABLE 2:** Child‐level characteristics at baseline and endline, by allocation group

Variable	Baseline *N* (%)			Endline *N* (%)		
	Control	Intervention	Total	Control	Intervention	Total
Total	466 (50.0)	467 (50.0)	932 (100)	440 (49.9)	441 (50.1)	881 (100)
Sex						
Male	210 (45.1)	206 (44.1)	416 (44·6)	202 (45.8)	161 (36.6)	363 (41.2)
Female	256 (54.9)	261 (55.9)	517 (55.4)	239 (54.2)	279 (63.4)	518 (58.8)
Age in months, mean (SD)	51.7 (9.7)	51.7 (11.4)	51.7 (10.6)	51.5 (11.2)	54.8 (11.7)	53.1 (11.6)
Disability						
No	420 (91.1)	409 (88.3)	829 (89.7)	413 (93.9)	414 (94.1)	826 (94.0)
Yes	41 (8.9)	54 (11.7)	95 (10.3)	27 (6.1)	26 (5.9)	53 (6.0)

### 3.3 Raw summary scores

Table 3 shows that at baseline, the intervention group had slightly higher mean prevalence of language delay and slightly lower mean prevalence of social delay. Mean prevalence of any delay was similar in the two groups.

**TABLE 3:** Raw summary statistics at baseline and endline, by allocation group

Time point	Group	CBCCs	Any delay		Language delay		Social delay	
		*N* (%)	Mean	SD	Mean	SD	Mean	SD
Baseline	Control	24	11.9%	7.0	3.9%	5.3	8.6%	5.1
	Intervention	24	11.4%	7.4	4.6%	4.7	7.9%	7.4
Endline	Control	22	10.0%	6.4	4.5%	4.3	7.3%	5.9
	Intervention	22	6.4%	6.0	2.5%	3.4	5.0%	5.3

By endline, mean prevalence of delay in the control CBCCs was higher than in the intervention group in all domains.

### 3.4 Primary analysis

Analysis of the difference between baseline and endline measures in the allocation groups shows a non‐significant reduction in delay associated with the study intervention across all domains (Table 4).

**TABLE 4:** Summary of differences observed between baseline to endline—linear regressions

	Any delay		Language delay		Social delay	
	Difference (95% CI)	*P* value	Difference (95% CI)	*P* value	Difference (95% CI)	*P* value
Primary analysis	−3.7 (−9.6 to 2.1)	0.21	−3.2 (−7.0 to 0.6)	0.09	−1.8 (−7.0 to 3.3)	0.48
Additional analyses with adjustment						
Number of caregivers	−3.1 (−9.0 to 2.9)	0.3	−3.6 (−7.5 to 0.4)	0.08	−0.8 (−5.7 to 4.2)	0.8
Total number of children	−3.9 (−9.8 to 2.1)	0.2	−3.3 (−7.2 to 0.6)	0.1	−1.9 (−7.1 to 3.3)	0.7
Has play/learning materials	−4.0 (−10.0 to 2.1)	0.2	−3.4 (−7.4 to 0.5)	0.09	−2.0 (−7.4 to 3.3)	0.5
Has an attendance register	−4.4 (−10.5 to 0.20	0.1	−3.3 (−7.3 to 0.7)	0.1	−2.6 (−7.8 to 2.6)	0.3
All the above	3.7 (−9.9 to 2.5)	0.2	−3.7 (−7.8 to 0.5)	0.08	−1.4 (−6.5 to 3.6)	0.6

Adjustment for baseline characteristics within the CBCCs showed little impact on the magnitude of the observed effect, and the difference remained non‐significant.

## 4 DISCUSSION

The data indicate no evidence of an effect of the intervention on the proportion of children observed to experience developmental delay in either or both of the language and social domains, at approximately 12 months after the intervention. Adjustment for a range of contextual variables does not impact this conclusion.

However, non‐significant differences in the reduction in delay were observed and need to be considered. A pronounced reduction in the language domain is particularly notable. The primary analysis suggests that the intervention may be associated with a reduction of 3.2% (95% CI indicating this may range from a reduction of 7.0% to an increase of 0.6%), over a period of 12 months. In the social domain, the intervention may be associated with a reduction of delay of 1.8% (95% CI −7.0% to 3.3%). These promising findings suggest that the intervention may have a positive effect on child development, particularly in the language domain, and warrant further investigation, with both a larger sample and longer follow‐up period to account for those limitations noted here.

Very few studies have been undertaken examining interventions to improve development outcomes of young children in similar settings as this. However, a recent four‐arm RCT in Malawi did examine CBCC caregiver training alongside parental education compared with caregiver training alongside financial incentives; caregiver training alone; and provision of learning materials (Ozler et al., 2016). After 18 months, it observed better development outcomes among the group that received caregiver training alongside parental education, with no changes in the other groups. Similar to our study, the authors also noted a particular improvement in the language domain, and they concluded that ‘although both classroom practice and parenting quality can be successfully manipulated through similar interventions, only the latter consistently caused improvements in children's language, cognitive and socio‐emotional skills in this context’.

The particularly pronounced change in the language domain may reflect the emphasis of the training programme on caregiver engagement of children in their local language, Chichewa, and creating opportunities for both spontaneous and formal speaking opportunities for all children. Such changes in practice are not dependent on external resources and would have been relatively simple for caregivers to implement on their return to their CBCCs. Having frequent access to a variety of reading materials such as big books and graded short story books in Chichewa to create a language rich environment for early years would increase children's acquisition of speaking and listening skills.

This study is among few to use an experimental design to evaluate an intervention with regard to its impact on child development outcomes within language acquisition and social development in a LMIC setting. However, the study is subject to several limitations that must be accounted for when interpreting the results. The study size, both in terms of sample—numbers of CBCCs and children—and duration were limited by available resources. A longer follow‐up period would have been useful to understand longer term effects of the intervention on development outcomes, including to understand the persistence of the effect. While it is possible that a larger sample would have yielded significant margins of error around the observed effect, it is not guaranteed. The closure of three CBCCs (one from the intervention arm and two from the control arm) between baseline and endline data collection is unfortunate, and while the closures may not be directly linked to the intervention or child development outcomes, they underline the precarious operating nature of CBCCs in this setting and the challenges facing caregivers to keep such centres open.

We were unable to verify whether those caregivers who received training continued to work at their CBCC for the entire duration of the follow‐up period or whether they ceased employment at some point. This scenario would lead to a dilution of the effect of the intervention, and thus, the observed difference may be an underestimate of any change that could have occurred given 100% retention.

There is a noticeable difference in the age and sex composition of children in the allocation groups at endline. Crude analyses (not reported) between the outcomes and sex, by allocation group and at both baseline and endline, indicate no significant association. However, we cannot rule out that an increase in older children or girls in the intervention CBCCs at endline is an unintended consequence of the intervention.

A short training programme such as this can only cover the most fundamental areas with limited time available to explore early literacy and numeracy skills. Encouraging caregivers to support children more flexibly requires more extensive training and opportunities to try out new approaches and to reflect on practice. The results of the other trial conducted in Malawi indicate that improvements in CBCC caregiver resourcing should be accompanied by support to parents. Community‐based ECD in Malawi holds tremendous potential for promoting children's development and learning in their earliest years, but further work and resources are required to ensure the quality required for to produce the desired child outcomes.

## CONFLICT OF INTEREST

The authors declare no competing interests.

## ETHICS STATEMENT

Ethical approval for this study was obtained from the National Committee on Research in the Social Sciences and Humanities, National Commission for Science and Technology, Malawi (P.02/16/83) and the University of Birmingham Ethics Committee, UK (ERN_15‐0048).

## Data Availability

The data that support the findings of this study are available in the UK Data Service ReShare repository at https://reshare.ukdataservice.ac.uk/853877/. The data are safeguarded and available on registration and reasonable request.
